# SARS-CoV-2 Early Screening at the Point of Entry: Travelers From Bangladesh to Italy–July 2020

**DOI:** 10.3389/fgene.2021.625607

**Published:** 2021-02-09

**Authors:** Martina Rueca, Antonino Di Caro, Cesare Ernesto Maria Gruber, Francesco Messina, Emanuela Giombini, Maria Beatrice Valli, Eleonora Lalle, Simone Lanini, Francesco Vairo, Maria Rosaria Capobianchi, Barbara Bartolini

**Affiliations:** National Institute for Infectious Diseases, INMI (Istituto Nazionale per le Malattie Infettive), “Lazzaro Spallanzani” IRCCS (Istituto di ricovero e Cura a Carattere Scientifico), Rome, Italy

**Keywords:** SARS-CoV-2, next generation genome sequencing, mutations, phylogenetic analysis, COVID-19, molecular epidemiology, early detection at point of entry

## Abstract

We report phylogenetic and mutational analysis by NGS of six SARS-CoV-2 strains from patients flying from Bangladesh to Italy (July 2020). Data suggest that no further circulation of such imported strains occurred in Italy, stating the efficacy of early screening at the point of entry and supporting the importance of molecular epidemiology in monitoring the efficacy of control measures.

## Introduction

The current outbreak of novel coronavirus (COVID-19) disease has spread across borders through travelers. Thanks to the lockdown measures, closure of unnecessary activities and services, as well as block of traveling from foreign countries was undertaken in Italy from 9th March to 3rd May. As a result, partial control of virus spread was achieved, and subsequently the country gradually returned activities to normal, including travel connections with other countries (ECDC., 2020). In the release phase, early detection of suspect cases at points of entry (POE), including ports, airports and ground crossings and implementation of appropriate control measures are crucial to reduce the risk for igniting new transmission chains (Alm et al., 2020; ECDC., 2020).

Here we report the phylogenetic and mutational analysis of SARS-CoV-2 strains harbored by travelers entering Italy from Bangladesh flying from Dhaka in early July 2020. The analyses were carried out on six samples randomly selected, and the data supported the importance of molecular epidemiology in achieving successful control of new infection waves from imported cases.

## Method

Nasopharyngeal-swabs (NPS) from 406 travelers coming from Dhaka on 2 airplanes landed in Rome Fiumicino airport were collected immediately upon disembarkment on July 7th, and sent to the Laboratory of Virology of the “L. Spallanzani” Institute, Rome, for SARS-Cov-2 diagnosis, resulting in 50 laboratory confirmed infections.

The presence of SARS-CoV-2 RNA in clinical samples was detected by a commercial RT-PCR assay [Cobas® SARS-CoV-2 (Roche Diagnostics)].

For sequence analysis, the full genome viral sequencing was performed for available residual samples from 6 patients involved in this outbreak. Nucleic acid extraction was performed by QiaSymphony automatic extractor, then Next Generation Sequencing (NGS) was carried out on Ion Torrent GSS5 platform using Ion AmpliSeq SARS-CoV-2 Panel, following manufacturer's instructions (ThermoFisher). Ethical approval for sequence analysis: no. 70/2018(17/12/2018).

Mean quality Phred score >20 raw reads were selected and trimmed using Trimmomatic software v.0.36 (Bolger et al., 2014). SARS-CoV-2 genomes were assembled using reference-based assembly method, with BWA v.0.7.12 (Li and Durbin, 2009) and Samtools v.1.3.1 (Li et al., 2009). Contigs were then verified using Geneious 2019.2.3. Single Nucleotide Variants (SNV) were called taking all mutations with a coverage ≥50 reads and a frequency >50%, and excluding mutations lying only in the first or last 5 nucleotides of the reads.

Sequences of SARS-CoV-2 strains from Italy and Bangladesh available at 5th October 2020 were retrieved from GISAID, selecting high coverage genomes. Sequences were clustered at 0.03% using CD-HIT v.4.6 software (Fu et al., 2012). Maximum likelyhood phylogenetic analysis was performed with IQ-TREE v.1.6.12, using Transition with invariable sites plus discrete Gamma model (TIM2+I+G) and 1,000 replicates; Wuhan-Hu-1 strain was adopted as phylogenetic outgroup (MN908947.3).

## Results

The phylogenetic lineage classification proposed by Rambaut et al. (2020) was used in the phylogenetic analysis, although maintaining, for comparison to previously published reports, also reference to clades reported in GISAID (Elbe and Buckland-Merrett, 2017).

**Figure 1:**
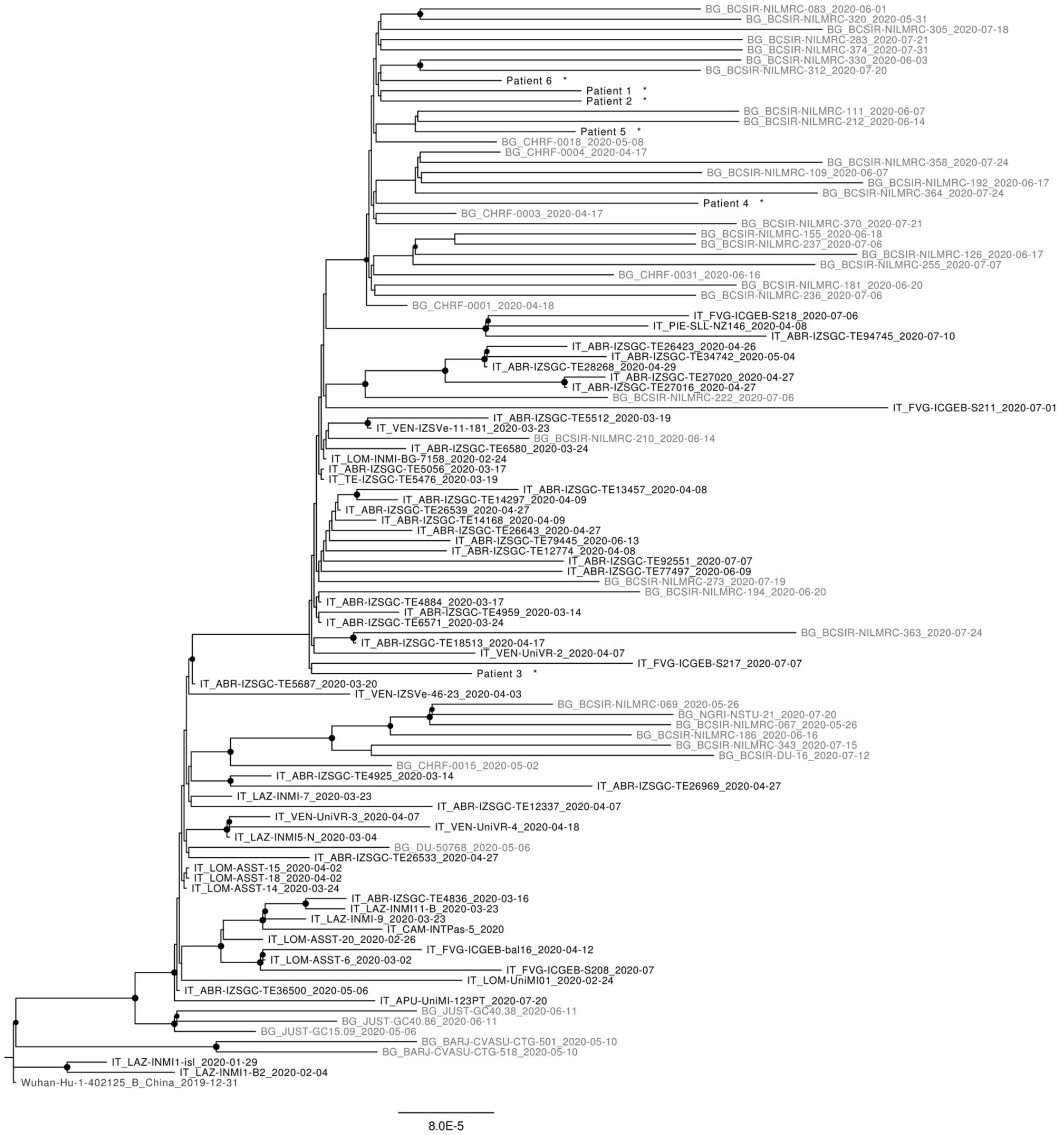
Maximum likelyhood phylogenetic tree of representative sequences from Italy (in black) and Bangladesh (in gray) available at 5th October 2020. All Nodes with bootstrap values >65% are highlighted. Sequence from this work are marked with *.

As can be seen in Figure 1, most sequences reported from Bangladesh, retrieved from the GISAID platform, form distinct clusters within B1 and B1.1 (G and GR) clades.

Concerning the study sequences from passengers flying from Bangladesh, all belong to B1.1 (GR) clade; 5 out of 6 of them fall within the Bangladesh-specific GR cluster highlighted by the phylogenetic analysis; the remaining sequence (sequence number = 3) is interspersed with other GR sequences of mixed origin, among which there are also sequences obtained in Italy in the same period.

Thirty-eight Single Nucleotide Variants (SNV), as compared to the reference strain Wuhan (Accession Number: MN908947), were observed in the study sequences: three in the Untranslated Regions (UTR), 15 synonimous and 20 non-synonimous (Table 1).

**Table 1:** Consensus sequences of study samples: differences vs. Wuhan-Hu-1sequence.

**Region**	**nt Position**	Ref. ^*^	**Patient 1**	**Patient 2**	**Patient 3**	**Patient 4**	**Patient 5**	**Patient 6**	**AA change**
5′UTR	241	C	T	T	T	T	T	T	–
Orf1ab	829	C				T			Syn
	1,163	A	T	T		T	T	T	I300F
	1,202	A			G				N313D
	3,037	C	T	T	T	T	T	T	Syn
	3,403	G	T						Syn
	4,331	C				T			Syn
	5,281	C			T				Syn
	5,800	G		T					L1845F
	5,869	C						T	Syn
	6,019	A	G						Syn
	7,743	G				T			S2493I
	8,026	A				G			Syn
	8,560	T		C					Syn
	9,136	G		T					M2957I
	10,626	C				T			A3454V
	11,083	G					T		L3606F
	11,719	G			A				Syn
	14,408	C	T	T	T	T	T	T	P4715L
	15,656	C				T			T5131I
	15,807	A						G	Syn
	17,427	G					T		Syn
	19,656	G		T					K6464N
S	23,403	A	G	G	G	G	G	G	D614G
	25,046	C		T					P1162S
	25,047	C					A		P1162C
	25,337	G	T						D1259Y
Orf3a	25,907	G				T			G172V
Orf7a	27,684	C					T		Syn
	27,707	C						T	A105V
N	28,083	G	T						Syn
	28,881	G	A	A	A	A	A	A	R203K
	28,882	G	A	A	A	A	A	A	R203K
	28,883	G	C	C	C	C	C	C	G204R
	29,272	C			T				Syn
	29,348	G				T			S359S
3'UTR	29,774	C					T		–
	29,779	G	T						–

* *Nucleotide positions are referred to Wuhan-Hu-1(reference genome MN908947)*.

*Nt, nucleotide; AA, amino acid; Syn, synonymous substitution; utr, untranslated region; Orf, open reading frame*.

More in details, all the strains carry a common set of 7 SNVs: C241T in 5′UTR and C3037T synonymous substitution in ORF1ab, that are the two most abundant mutations found in Bangladesh sequences and often found simultaneously, according to Ahmed Shishir et al. (2020). 14408 C>T and 23403 A>G are two additional non-synonimous mutations, often found simultaneously, leading to P4715L in ORF1ab and D614G changes in Spike protein (the signature mutation for G clade). Finally, the SNVs 28881 G>A, 28882 G>A and 28883 G>C, leading to R203K and G204R changes in Nucleocapsid protein.

Interestingly the non-synonimous SNV 1163 A>T (I300F) in ORF1 is detected in all sequences here described except in patient 3, that is not included in the Bangladesh-specific GR cluster.

## Discussion

As emerged from a previous study, a high percentage of virus sequences isolated in India and Bangladesh are closely related to European and US sequences carrying the mutations typical of the G clade (D614G in S and P4715L in Nsp12) (Islam et al., 2020). Moreover, the sequences reported here carry some of the mutations highly prevalent in Bangladesh sequences available on GISAID, among which the I300F mutation (Ahmed Shishir et al., 2020). This is found to affect the structural stability of Nsp2 (metyltransferase like domain), modulating host cell survival strategy (Ahmed Shishir et al., 2020), and deserves further attention.

Since the phylogenetic tree includes all the sequences available by the 5th October from Bangladesh and Italy (0.2% of positive cases in Italy were sequenced at that time), the confinement of most (*n* = 5) sequences within the Bangladesh-specific GR cluster suggests effective containment and despite a limit of this study is represented by the limited number of genomes analyzed (12% of positive cases detected in two flights), no further circulation of virus imported from this country occurred after its importation on 7th of July to Rome.

In a context where travel and business relationships between different countries/continents favor the spread of the virus, sequencing and phylogenetic analysis allows to clearly recognize the cluster of imported sequences, showing strong genetic links with other sequences from the country of origin, and no further circulation in the country of destination. Therefore, this data show how sequencing of whole-genome of SARS-CoV-2 and phylogenetic analysis are of great support to molecular epidemiology. These results additionally may provide accurate information about the fate of imported viral strains of the virus, hence inform about the efficacy of implemented control measures.

## Data Availability Statement

The datasets presented in this study can be found in online repositories. The names of the repository/repositories and accession number(s) can be found at: https://www.gisaid.org/, EPI_ISL_590693; https://www.gisaid.org/, EPI_ISL_590694; https://www.gisaid.org/, EPI_ISL_590695; https://www.gisaid.org/, EPI_ISL_590696; https://www.gisaid.org/, EPI_ISL_590697; https://www.gisaid.org/, EPI_ISL_590698.

## Ethics Statement

The studies involving human participants were reviewed and approved by ethics committee of INMI (Ethical approval: no. 70/2018(17/12/2018). Written informed consent for participation was not required for this study in accordance with the national legislation and the institutional requirements.

## Author Contributions

BB and MR: conceptualization. MR, MV, and EL: methodology. CG and FM: software. CG and EG: formal analysis. MR: investigation. FV and SL: resources. MR: writing—original draft preparation. BB and MC: writing—review and editing. CG: visualization. BB: supervision. MC and AD: project administration and funding acquisition. All authors have read and agreed to the published version of the manuscript.

## Conflict of Interest

The authors declare that the research was conducted in the absence of any commercial or financial relationships that could be construed as a potential conflict of interest.
